# Identification and tissue-specific expression of rutin biosynthetic pathway genes in *Capparis spinosa* elicited with salicylic acid and methyl jasmonate

**DOI:** 10.1038/s41598-020-65815-2

**Published:** 2020-06-01

**Authors:** Farzad Kianersi, Mohammad Reza Abdollahi, Asghar Mirzaie-asl, Dara Dastan, Faiza Rasheed

**Affiliations:** 1 Department of Agronomy and Plant Breeding, Faculty of Agriculture, Bu-Ali Sina University, Hamedan, Iran; 2 Department of Plant Biotechnology, Faculty of Agriculture, Bu-Ali Sina University, Hamedan, Iran; 3 Medicinal Plants and Natural Products Research Center, Hamadan University of Medical Sciences, Hamadan, Iran; 4 Department of Pharmacognosy and Pharmaceutical Biotechnology, School of Pharmacy, Hamadan University of Medical Sciences, Hamadan, Iran; 5 Department of Plant Breeding, Swedish University of Agricultural Sciences, Växtskyddsvägen 1, SE-230 53 Alnarp, Sweden; 6 KTH Royal Institute of Technology, School of Chemical Science and Engineering, Fibre and Polymer Technology, SE-100 44 Stockholm, Sweden

**Keywords:** Biotechnology, Molecular biology, Plant sciences

## Abstract

*Capparis spinosa* is an edible medicinal plant which is considered as an excellent source of rutin. Rutin is a glycoside of the flavonoid quercetin that has been reported to have a beneficial role in controlling various diseases such as hypertension, arteriosclerosis, diabetes, and obesity. In this study, the partial cDNA of four genes involved in the rutin biosynthetic pathway including 4-coumaroyl CoA ligase (4CL), flavonoid 3′-hydroxylase (F3′H), flavonol synthase (FLS) and flavonol-3-O-glucoside L-rhamnosyltransferase (RT) were identified in *C.spinosa* plants for the first time. The protein sequences of these genes shared high similarity with the same proteins in other plant species. Subsequently, the expression patterns of these genes as well as rutin accumulation in *C.spinosa* leaves treated with different concentrations of salicylic acid (SA) and methyl jasmonate (MeJA) and also in different tissues of Caper plants treated with 100 mgL^−1^ SA and 150 μM MeJA were evaluated. The expression of all four genes was clearly up-regulated and rutin contents increased in response to MeJA and SA treatments after 24 h. The highest rutin contents (5.30 mgg^−1^ DW and 13.27 mgg^−1^ DW), as well as the highest expression levels of all four genes, were obtained using 100 mgL^−1^ SA and 150 μM MeJA, respectively. Among the different tissues, the highest rutin content was observed in young leaves treated with 150 μM MeJA, which corresponded to the expression of related genes, especially RT, as a key gene in the rutin biosynthetic pathway. These results suggest that rutin content in various tissues of *C. spinosa* can be enhanced to a significant extent by MeJA and SA treatments and the gene expression patterns of rutin-biosynthesis-related genes are regulated by these elicitors.

## Introduction

Flavonoid biosynthetic pathway in higher plants has been well defined and several key enzymes involved in this pathway have been identified^1–3^. Rutin is one of the most important flavonoids and plays a key role to protect plants against ultraviolet radiation or pathogens and is also used to prevent the side effects of some diseases such as cancer treatments, diabetes, and hypercholesteremia^4,5^. The presence of rutin has been reported in many plant species, but only a limited number of plants such as *Fagopyrum esculentum*^6,7^ and Cappris species, especially, *C. spinosa*^8^ are identified as the biggest plant sources of rutin (or rutoside). *C. spinosa* L. (Capparidaceae) an aromatic plant, is widely distributed in the coastal areas of the Mediterranean basin and is reported with a 2.8% of rutin content in the leaf tissue^9^.

**Figure 1:**
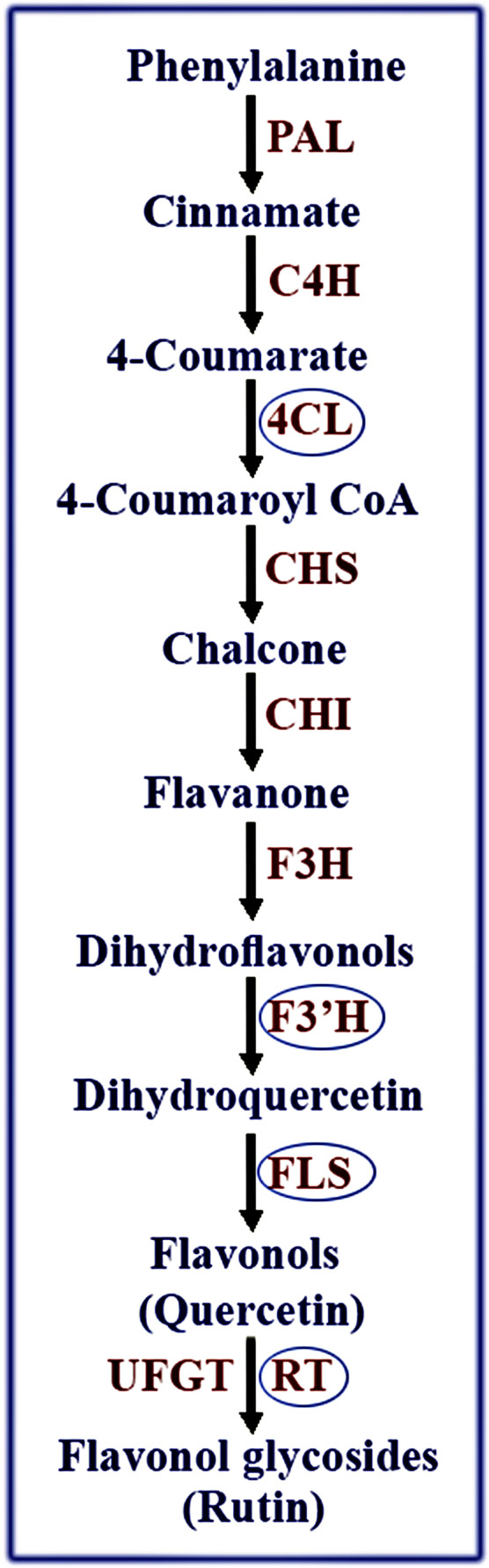
The biosynthetic pathway of rutin. Enzyme genes that have been identified in *Capparis spinosa*, are marked with a blue line around them. Phenylalanine ammonium lyase (PAL), cinnamic acid 4-hydroxylase (C4H), 4-coumarate-CoA ligase (4CL), chalcone synthase (CHS), chalcone isomerase (CHI), flavanone-3-hydroxylase (F3H), flavonoid-3′-hydroxylase (F3′H), flavonol synthase (FLS), flavonoid 3-O-glucosyltransferase (UFGT), flavonol-3-O-glucoside L-rhamnosyltransferase (RT).

The main genes involved in rutin biosynthetic pathway are phenylalanine ammonium lyase (PAL), cinnamate-4-hydroxylase (C4H), 4-coumarate-CoA ligase (4CL), chalcone synthase (CHS), chalcone isomerase (CHI), flavanone-3-hydroxylase (F3H), flavonoid-3′-hydroxylase (F3′H), flavonol synthase (FLS), flavonoid 3-O-glucosyltransferase (UFGT) and flavonol-3-O-glucoside L-rhamnosyltransferase (RT) (Fig. 1). Some of them including PAL, C4H, 4CL, CHI, FLS, F3H, F3′H and RT are identified and isolated from Tartary buckwheat (*Fagopyrum tataricum*) and common buckwheat (*Fagopyrum esculentum*)^2,3,10^. However, rutin biosynthetic pathway genes are not characterized in the caper plant yet.

**Figure 2:**
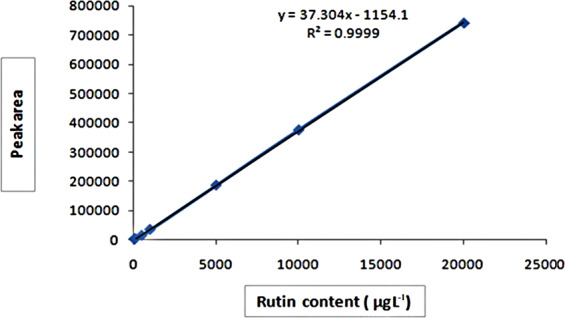
Rutin standard curve determined by HPLC. Rutin standard curve was used for linear regression equations: Y = 37.304X+1154.1(R^2^ = 0.9999, Y: peak area, X: rutin content).

**Figure 3:**
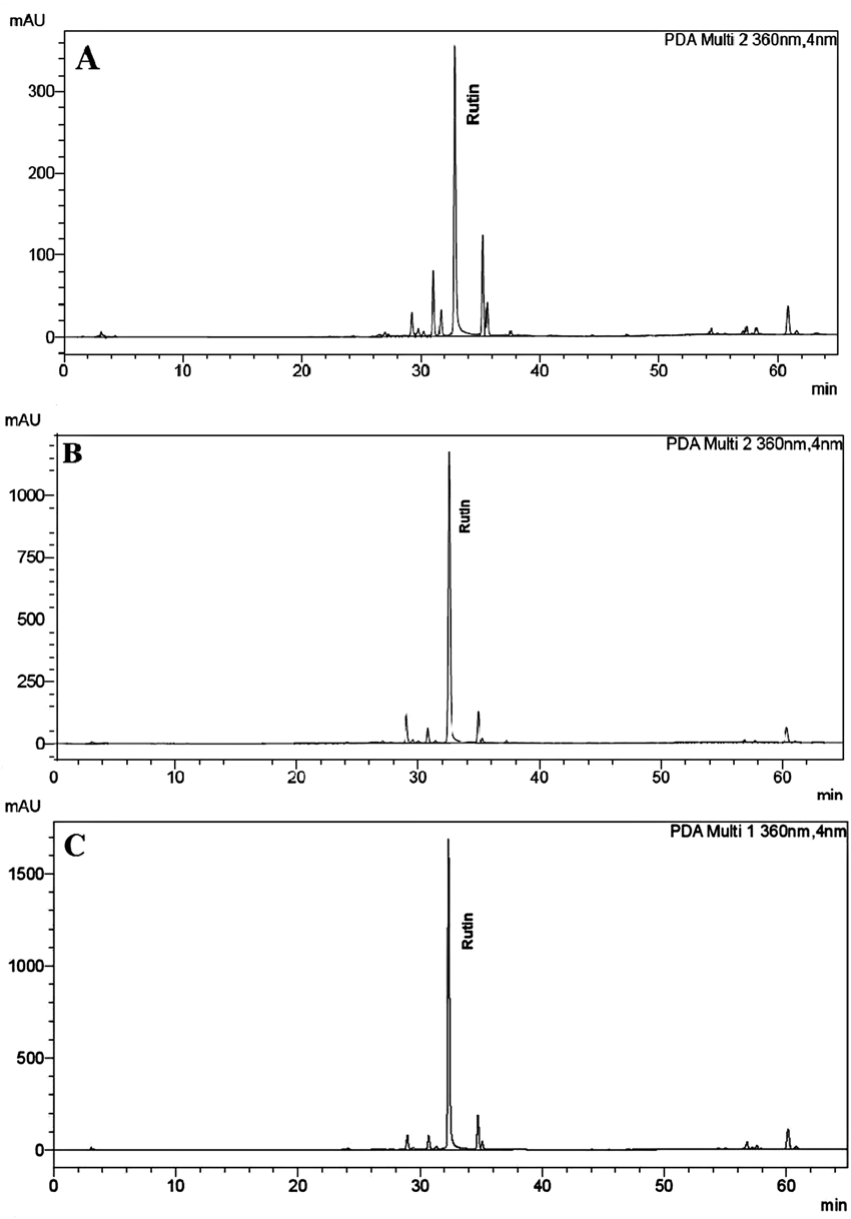
Peak area detected by HPLC for rutin in (**A**) Non-treated leaves, (**B**) SA-treated leaves and (**C**) MeJA-treated leaves.

Chemical elicitors often induce the biosynthesis of secondary metabolites by upregulating their biosynthetic pathways genes. Several chemical stimulants such as jasmonic acid (JA), methyl jasmonate (MeJA), salicylic acid (SA), ethylene, and heavy metals have been used in elicitation studies^11^. MeJA is a linolenic acid derivative that can induce plant defense responses to biotic and/or abiotic stresses and it is widely used exogenously in plant cell cultures to activate secondary metabolites’ production^11^. MeJA triggered enhancement of the content of many secondary metabolites such as paclitaxel, phenols and flavonoids in different plant species has been well documented by various studies^12–15^. JA and MeJA play a key role in a signal transduction pathway and can regulate defense genes in plants. The previous studies showed that genes involved in jasmonate biosynthesis, secondary metabolism, cell wall formation, and stress tolerance are up-regulated by MeJA elicitation^12^. Besides this, SA as a signal molecule is involved in many physiological and biochemical processes within plants^16^. SA and its analogues are used comparatively lesser than MeJA for elicitation studies^17,18^. Like MeJA, SA also induces many plant secondary metabolites such as isoflavonoids, terpenes, phytosterols and phenylpropanoids^19^ and plays an important role in plant response to a variety of environmental stresses^20–22^.

Keeping the pharmacological and economic value of rutin and its high content in *C*. spinose^8^, the study was designed to present an efficient alternate source of rutin. Moreover, for the sustainable production of rutin in plant various chemical elicitors were employed and the pattern of the regulation of genes involved in rutin biosynthetic pathway was evaluated.

## Results

### Effect of elicitors on rutin content of *C. spinosa* leaves

**Figure 4:**
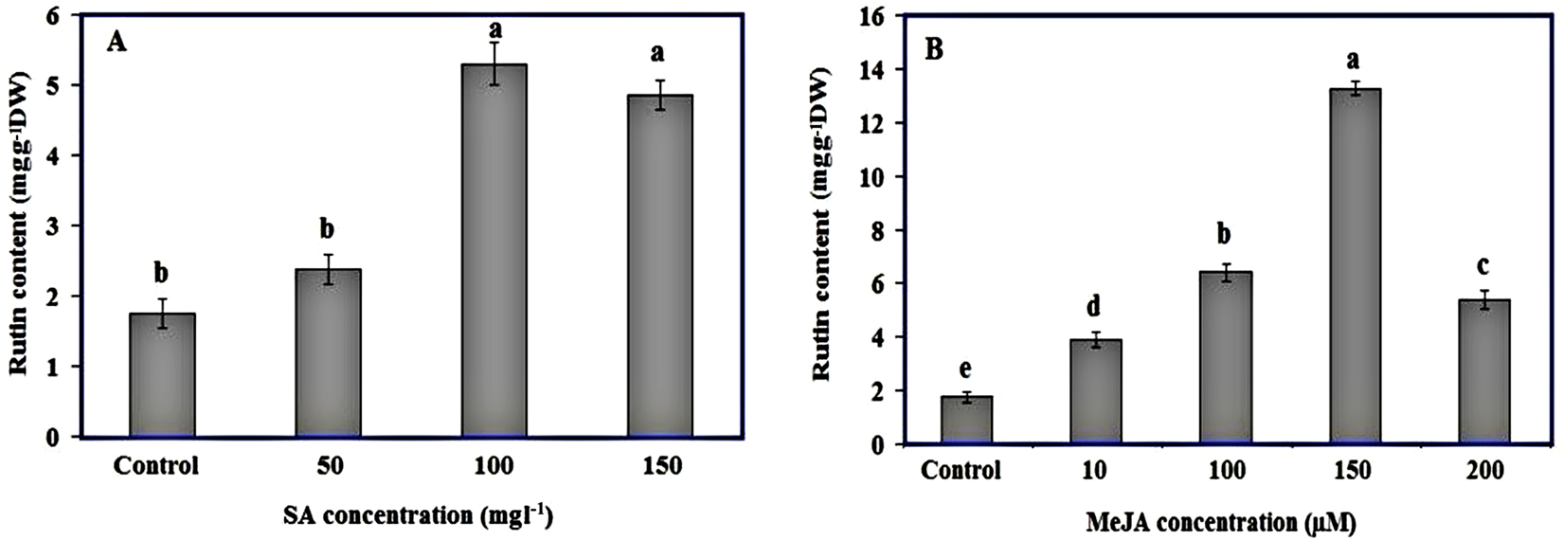
Rutin contents in leaves of *Capparis spinosa* plants treated with different concentrations of (**A**) Salicylic acid and (**B**) Methyl jasmonate. Bars with different letters are significantly (P < 0.05) different according to Duncan’s test. Error bars indicate standard error values.

The effect of MeJA and SA treatments on rutin contents is described in Fig. 4A,B. Significant differences (p < 0.05) were observed in rutin contents of Caper leaves treated with different concentrations of both SA and MeJA compared to the control plants after 24 h. The rutin content was elevated by SA treatments and reached the highest point at 100 mgL^−1^ SA (5.30 mgg^−1^ DW) (Fig. 4A). In detail, the rutin contents of Caper leaves treated with 100 and 150 mg l^−1^ SA were 5.30 and 4.85 mgg^−1^ DW, respectively, that were 3 and 2.75 fold higher than those found in the control leaves (Fig. 4A). However, SA treatment at 50 mgL^−1^ had no significant effect on rutin content of Caper leaves. Also, exogenous application of MeJA treatments significantly enhanced the rutin contents in leaves of treated plants compared to control plants. Rutin content of Caper plants treated with 10 and 100 μM MeJA were significantly increased up to 3.89 and 6.39 mgg^−1^ DW, that were 2.22 and 3.65-fold higher than control plants (1.75 mgg^−1^ DW), respectively and peaked at 150 μM MeJA (13.27 mgg^−1^ DW, 7.58 times of control plant). Application of 200 μM MeJA significantly increased the rutin contents to 5.39 mgg^−1^ DW (3-fold higher than control) (Fig. 4B).

### Rutin contents in various tissues of Caper plants treated with 150 μM MeJA and 100 mgL^−1^ SA

**Figure 5:**
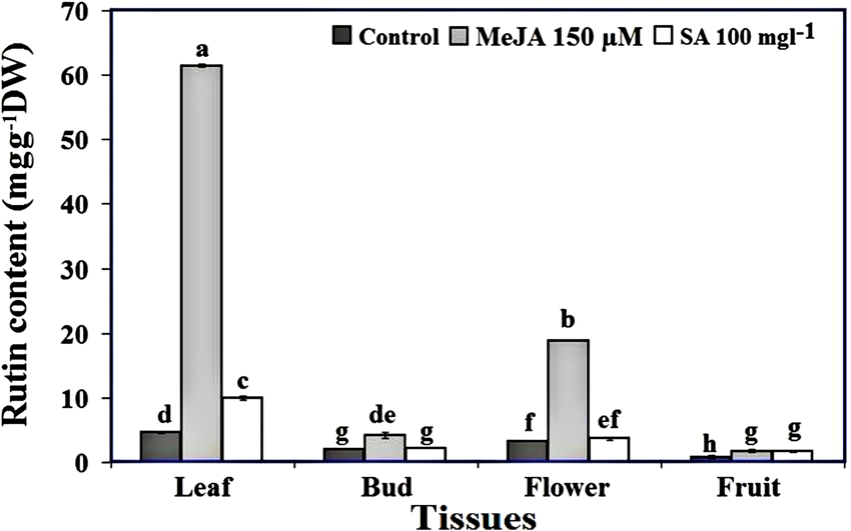
Rutin Contents in different tissues of *Capparis spinosa* treated with 150 μM methyl jasmonate and 100 mgL^−1^ Salicylic acid. Bars with different letters are significantly (P < 0.05) different according to Duncan’s test. Error bars indicate standard error values.

The amount of rutin in various tissues of the Caper plants treated with 150 μM MeJA and 100 mgL^−1^ SA is shown in Fig. 5. Various tissues of Caper plants showed different responses to MeJA and SA treatments with respect to rutin levels. The amount of rutin in the young leaves (4.48 mgg^−1^ DW) of the control plants was significantly higher than those of other tissues in these plants. The lowest amount of rutin was observed in the fruits (0.83 mgg^−1^ DW) of control plants (Fig. 5). Exogenous application of MeJA at concentration of 150 μM significantly increased the rutin contents in all tissues of the plant compared to their control. The highest amount of rutin was observed in the young leaves (61.46 mgg^−1^ DW), which was several-fold higher than those of its control and other treatments (Fig. 5). The fruits of Caper treated with 150 μM MeJA showed the lowest content of rutin (1.77 mgg^−1^ DW) compared with other tissues of these plants (Fig. 5). Spraying of Caper plants with 100 mgL^−1^ SA significantly increased rutin levels in young leaves (9.99 mgg^−1^ DW) compared to control plants, while there was no significant increase in rutin content of other tissues in treated plants than control plants (Fig. 5).

### Gene isolation and sequence analysis

**Figure 6:**
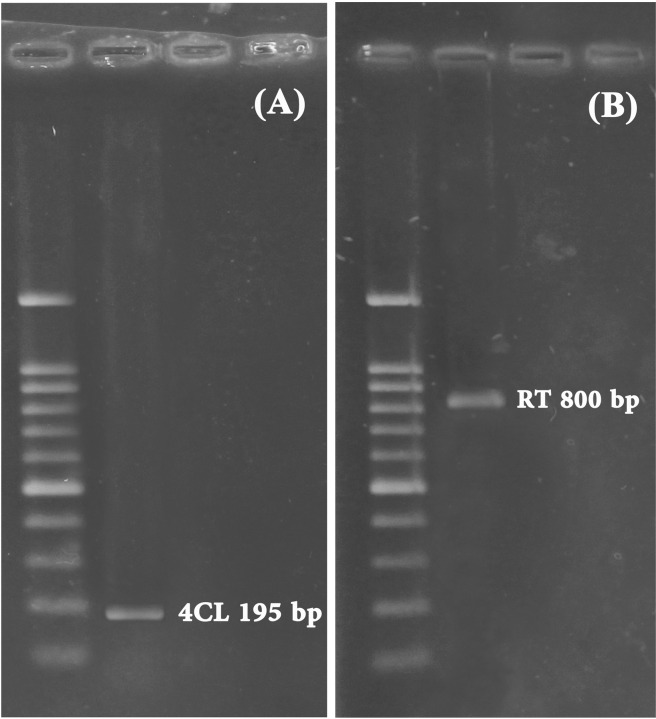
Agarose gel electrophoresis of PCR assays for the gene identification: (**A**) Partial sequence of 4-coumarate-CoA ligase (4CL) gene; 195 bp, (**B**) Partial sequence of Flavonol-3-O-glucoside L-rhamnosyltransferase (RT) gene; 800 bp.

The partial sequences of two genes involved in the rutin biosynthetic pathway including 4Cl and RT were identified from *C. spinosa* plants for the first time. These genes encode the first and last important enzymes involved in rutin biosynthetic pathway. Also, we found two partial cDNA segments of F3′H and FLS genes with 101 bp, from PRJNA311285 *Capparis spinosa* transcriptome sequencing project by blast analysis of these sequences with the same genes in other plants (Table 1). These genes encode the middle enzymes of rutin biosynthetic pathway. The amino acid sequences encoded by partial cDNAs for 4CL (GenBank accession number, MK301444), F3H, FLS, and RT (GenBank accession number, MK301445) were aligned and compared to orthologous sequences from other plants. The 4CL partial cDNA, 195 bp in length (Fig. 6A), encoded a protein of 65 amino acids, sharing 63–66% identity with 4CL genes from *Camelina sativa*, *Eutrema salsugineum*, *Raphanus sativus*, *Nelumbo nucifera*, *Tarenaya hassleriana*, *Capsella rubella*, *Arabidopsis lyrata* and *Brassica napus* (Table 1). The F3′H partial cDNA sequence with 101-bp length, encoded a protein of 33 amino acids, sharing 88–91% identity with F3′H genes from *Matthiola incana*, *R. sativus*, *B. napus*, *Brassica rapa*, *E. salsugineum*, *Brassica oleracea*, *C. sativa* and *F. esculentum* (Table 1). Similarly, the 101-bp FLS partial cDNA sequence encoded a protein of 33 amino acids, sharing 82–91% identity with those from *Parrya nudicaulis*, *C. sativa*, *Lactuca sativa*, *Ipomoea purpurea*, *Ziziphus jujuba*, *Morus notabilis* and *Jatropha curcas* (Table 1). Also, the length of RT partial cDNA was 800 bp, (Fig. 6B) and the encoded protein contained 266 amino acids. BLAST analysis showed that this protein shared 60–69% identity with RT from *T. hassleriana*, *C. rubella*, *J. curcas*, *Herrania umbratica*, *Arabidopsis thaliana*, *Hevea brasiliensis* and *Gossypium hirsutum* (Table 1).

**Table 1:** Alignment of 4-coumaroyl CoA ligase (4CL), flavonoid 3′-hydroxylase (F3′H), flavonol synthase (FLS) and flavonol-3-O-glucoside L-rhamnosyltransferase related amino acid sequences in *Capparis spinosa* with other plants.

Genes (Description)	Species	E-value	Sequence identity (%)	GenBank Numbers
4CL (4-coumarate-CoA ligase)	*Camelina sativa*	6e-23	65%	XP_010484070.1
	*Eutrema salsugineum*	6e-23	65%	XP_024007548.1
	*Camelina sativa*	7e-23	65%	XP_010460206.1
	*Raphanus sativus*	1e-22	66%	XP_018441958.1
	*Nelumbo nucifera*	2e-22	65%	XP_010250796.1
	*Tarenaya hassleriana*	3e-22	65%	XP_010520611.1
	*Capsella rubella*	4e-22	63%	XP_006280253.1
	*Arabidopsis lyrata*	5e-22	63%	XP_002866551.1
	*Brassica napus*	7e-22	66%	XP_013701364.1
F3′H (Flavonoid-3′-hydroxylase)	*Matthiola incana*	5e-18	91%	AAG49301.1
	*Raphanus sativus*	5e-18	91%	BAX90120.1
	*Brassica napus*	6e-18	91%	XP_013715260.1
	*Brassica rapa*	7e-18	91%	ABY89687.1
	*Eutrema salsugineum*	7e-18	88%	XP_006399285.1
	*Brassica oleracea*	7e-18	91%	XP_013606999.1
	*Camelina sativa*	1e-17	88%	XP_010423152.1
	*Fagopyrum esculentum*	2e-16	88%	ADT63065.1
FLS (Flavonol synthase)	*Parrya nudicaulis*	6e-15	91%	ADY02821.1
	*Camelina sativa*	7e-15	91%	XP_010484104.1
	*Parrya nudicaulis*	7e-15	91%	ADY02802.1
	*Lactuca sativa*	1e-14	82%	XP_023751792.1
	*Lactuca sativa*	1e-11	85%	BAG12186.1
	*Ipomoea purpurea*	2e-14	88%	AFP53278.1
	*Ziziphus jujuba*	2e-14	88%	XP_015879930.1
	*Morus notabilis*	3e-14	91%	XP_010092364.1
	*Jatropha curcas*	3e-14	88%	XP_012078184.1
RT (Flavonol-3-O-glucoside L-rhamnosyltransferase)	*Tarenaya hassleriana*	1e-127	69%	XP_010537036.1
	*Capsella rubella*	1e-117	61%	XP_006281795.1
	*Jatropha curcas*	3e-116	61%	XP_012074195.1
	*Herrania umbratica*	2e-115	62%	XP_021288606.1
	*Arabidopsis thaliana*	2e-115	60%	NP_200212.1
	*Hevea brasiliensis*	4e-115	63%	XP_021677578.1
	*Gossypium hirsutum*	4e-115	61%	XP_016702871.1
	*Arabidopsis thaliana*	3e-112	60%	BAE98680.1
	*Arabidopsis thaliana*	6e-112	60%	AAM65481.1

**Figure 7:**
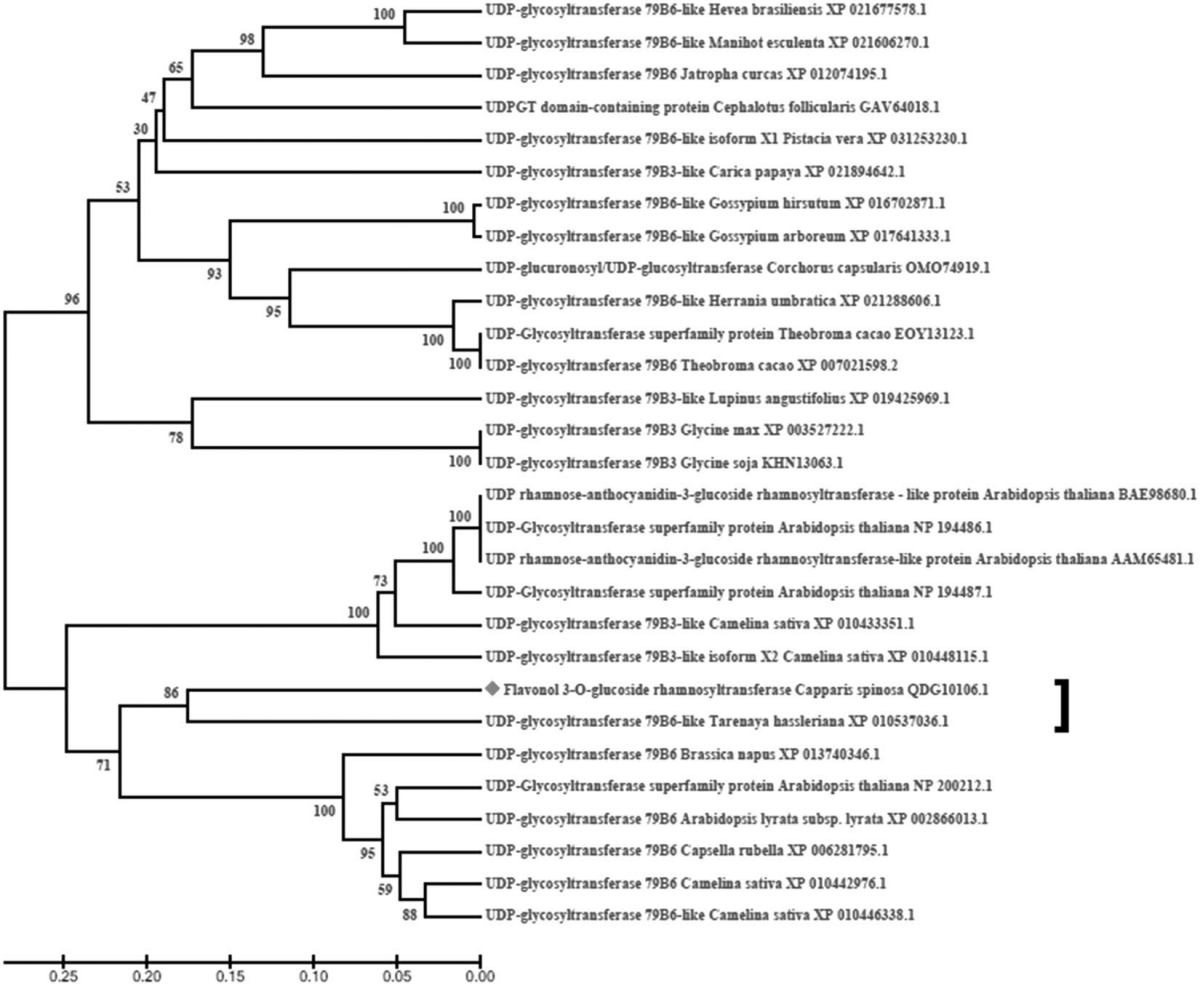
Molecular phylogenetic tree inferred from the deduced amino acid sequences of flavonol-3-O-glucoside L-rhamnosyltransferase (RT) from *Capparis spinosa* and related UDP-glycosyltransferases from other plant species. A molecular phylogenetic tree was constructed by the UPGMA method with 1000 bootstrap values using MEGA7 software. Bar indicates 0.05 amino acid substitutions per site.

A phylogenetic analysis was also performed using the deduced amino acid sequence of RT from the *C. spinosa* with the corresponding UDP-glycosyltransferases from other plant species (Fig. 7). The amino acid sequence of RT from *C.spinosa* showed the closest relationship with UDP-glycosyltransferase 79B6-like from the *T. hassleriana*, a plant from the Brassicaceae sister family, the Cleomaceae (Fig. 7). Also, the RT amino acid sequence, identified in this study, and most of the UDP-glycosyltransferases from other plant species of brassicaceae family grouped together and formed brassicales-specific clade (Fig. 7). Since Caper is also a plant of the brassicales order, this result can support the identification of the RT gene in this plant.

As shown in Fig. 1, two key enzymes, named flavonol-3-O-glucoside L-rhamnosyltransferase (RT) and flavonol 3-O-glucosyltransferase (UFGT), are involved in the production of rutin from quercetin. The sequence identified in this study is similar to both of these genes and both are in the UDP-glycosyltransferase group. In this study, we proved that the identified sequence is more similar to RT than UFGT. Based on the NCBI database, the proteins sequence of the RT and UFGT enzymes for the *G. hirsutum*, *Glycine max* and *A. thaliana* were found. Then, the partial sequence of RT, identified in this study, was blasted with both enzymes in each species. Our sequence showed the more identity with RT than UFGT in all three species (Table 2).

**Table 2:** Alignment of Flavonol-3-O-glucoside L-rhamnosyltransferase (RT) related amino acids in *Capparis spinosa* with Flavonol-3-O-glucoside L-rhamnosyltransferase and flavonoid-3-O-glucosyltransferase amino acids from *Gossypium hirsutum*, *Glycine max* and *Arabidopsis thaliana*.

Genes (**Description)**	Species	Query cover	E value	Sequence identity	GenBank numbers
Anthocyanidin 3-O-glucosyltransferase-like (LOC107912543) or Flavonol-3-O-glucoside L-rhamnosyltransferase, mRNA	*Gossypium hirsutum*	89%	2e-55	38%	XP_016696248.1
Flavonoid 3-O-glucosyltransferase-like (LOC107899989), mRNA	*Gossypium hirsutum*	50%	2e-05	25%	XP_016681210.1
Flavonol 3-O-glucoside (1->6) rhamnosyltransferase	*Glycine max*	98%	5e-66	41%	BAN91401.1
Flavonol 3-O-glucosyltransferase UGT89B1	*Glycine max*	89%	2e-11	24%	XP_003528248.1
UDP rhamnose-anthocyanidin-3-glucoside rhamnosyltransferase - like protein	*Arabidopsis thaliana*	100%	1e-118	60%	BAE98680.1
Putative flavonol 3-O-glucosyltransferase	*Arabidopsis thaliana*	68%	1e-09	24%	AAC35238.1

### Expression analysis of rutin related-genes in leaves of Caper plants treated with different concentrations of MeJA and SA

**Figure 8:**
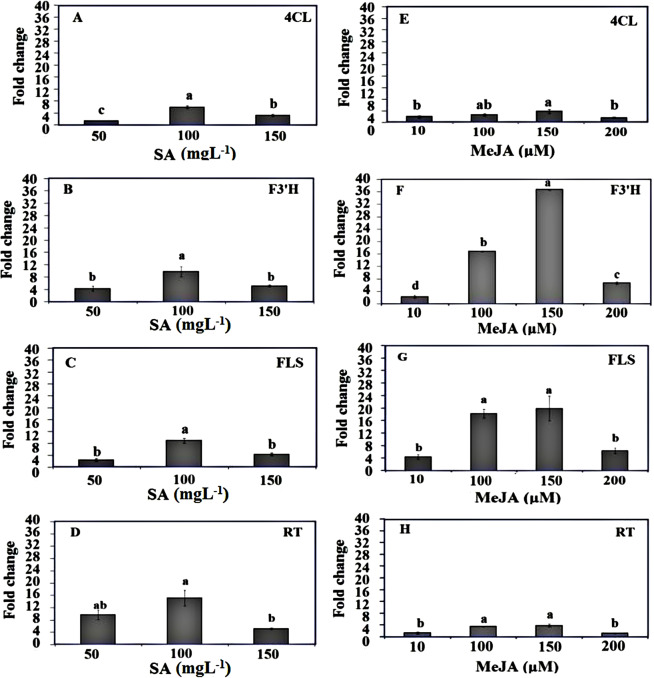
Expression levels of rutin biosynthesis-related genes in *Capparis spinosa* leaves under exogenous application of SA (**A–D**) and MeJA (**E–H**) treatments. Transcription levels for each gene was normalized based on the Ct values of Ubiquitin as a reference gene. Bars with different letters are significantly (P < 0.05) different according to Duncan’s test. Error bars indicate standard error values.

The mRNA transcript levels of some genes involved in rutin biosynthesis were investigated by qRT-PCR in leaves of Caper plants under exogenous application of SA and MeJA, 24 h after initial treatment (Fig. 8). The transcript levels were higher in MeJA and SA treated plants compared to the untreated controls for all studied genes. The transcript levels were highest at 100 mgL^−1^ SA treatment for all of the genes; that is, the transcript levels for 4CL, F3′H, FLS and RT were 5.89, 9.74, 8.87 and 15.90-fold higher, than that for the none-treated plants, respectively (Fig. 8A–D). In detail, the expression of 4CL, F3′H, FLS and RT genes increased to 1.32, 4.21, 2.36 and 9.66 times of control plants at 50 mgL^−1^ SA, respectively, and further increased to 5.89, 9.74, 8.87 and 15.90 times of the untreated control at 100 mgL^−1^ SA treatment, and then significantly decreased at 150 mgL^−1^ SA, while still maintained the 3.20, 5.11, 4.30 and 5.13-folds higher level than the control, respectively (Fig. 8A–D).

Also, a differential expression pattern was observed for the four rutin biosynthesis related genes, when treated with different concentrations of MeJA. In detail, the expression of 4CL and F3′H genes were gradually increased at 10 μM (1.90 and 2.28,-fold higher than control, respectively) and 100 μM (2.56 and 16.90,-fold higher than control, respectively) MeJA treatments and peaked at 150 μM that were 3.70 and 36.73-fold higher than control plants, respectively (Fig. 8E,F). Also, the expression levels of FLS and RT genes were 4.32 and 1.33-fold higher than control plants when treated with 10 μM MeJA and significantly increased to 18.20 and 3.54 times of controls at 100 μM MeJA treatment. The expression levels of these genes peaked at 150 μM MeJA treatment, showed 19.86 and 3.53-fold higher levels of expression rather than controls, respectively (Fig. 8G,H). It should be noted that there was no statistically significant differences between 100 μM and 150 μM MeJA levels for expression of FLS and RT genes (Fig. 8G,H). The transcripts levels of all genes were significantly decreased at 200 μM MeJA, while the expression levels were still much higher than control plants in this treatment (Fig. 8E–H).

### Expression analysis of rutin related-genes in different tissues of Caper plant treated with 150 μM MeJA and 100 mgL^−1^ SA

**Figure 9:**
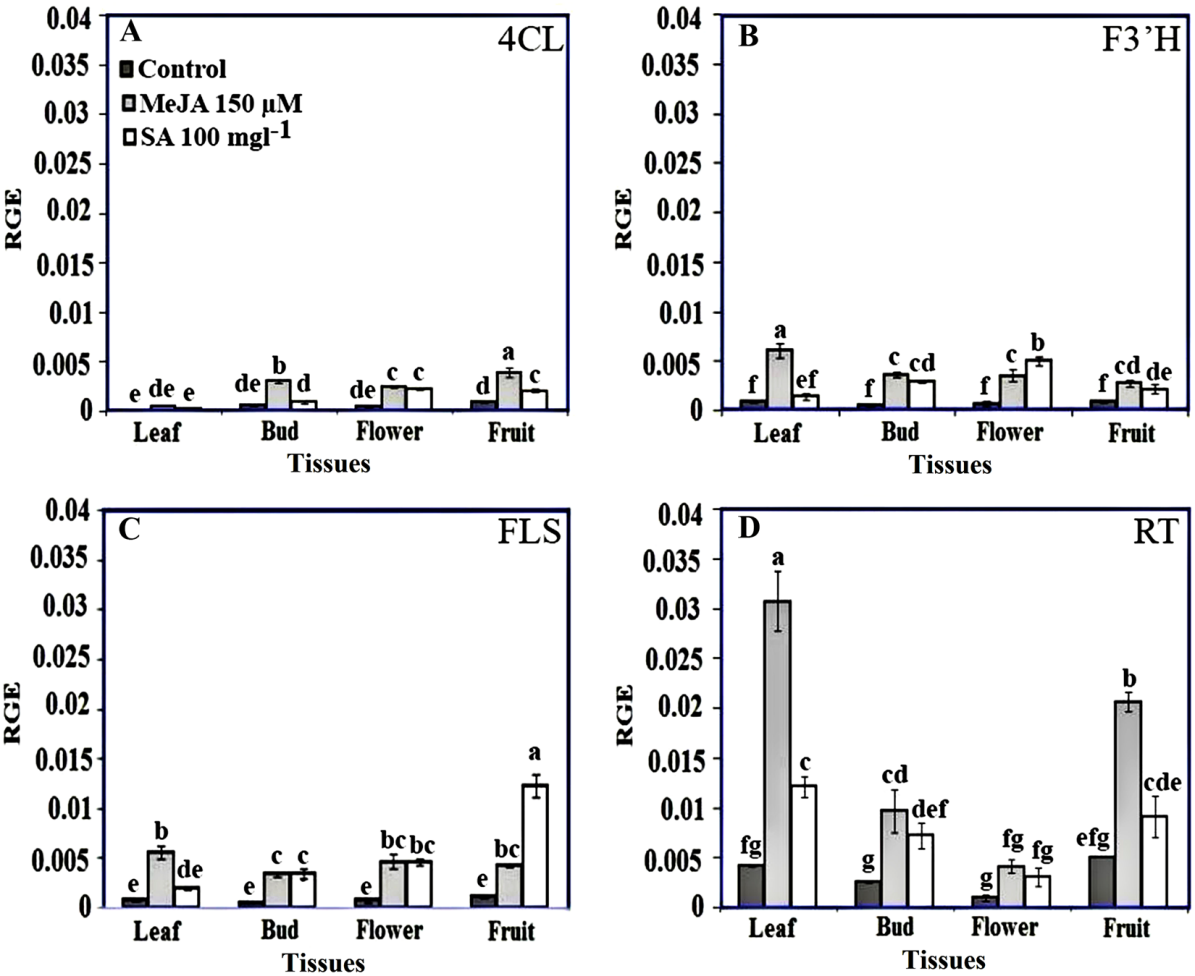
Relative gene expression (RGE) of early, middle and last pathway genes of rutin biosynthesis including 4CL, F3′H, FLS and RT in different tissues of control (untreated) and MeJA- and SA-treated Caper plants. Real-time qPCR was based on the Ct values. The Ct value for each sample was normalized using the reference gene Ubiquitin. Bars with different letters are significantly (P < 0.05) different according to Duncan’s test. Error bars indicate standard error values.

To investigate the mechanism controlling rutin biosynthesis in different tissues of Caper plants, the expression levels of four identified genes were investigated in various tissues of the control plants and also treated plants with 150 μM MeJA and 100 mgL^−1^ SA using qRT-PCR method. Different plant tissues, as well as different treatments of MeJA and SA, showed significant differences in expression of the studied genes (Fig. 9A–D). In the control plants, the highest expression of the 4CL gene was observed in fruits (9.46 × 10^−4^), which was significantly higher than the expression of this gene in young leaves tissues. In Caper plants treated with 150 μM MeJA, the expression of the 4CL gene was increased significantly in the bud, flower and fruit tissues compared to the same tissues in the control plants. The highest expression of the 4CL gene was observed in the fruits (3.84 × 10^−3^) of plants treated with MeJA (Fig. 9A). Also, the amount of 4CL gene expression in flower and fruit of SA-treated plants showed a significant increase compared to their control and the same treatment in other tissues of the plant (Fig. 9A). The lowest expression of 4CL gene was observed in young leaves of control (1.67 × 10^−4^) and SA-treated (2.83 × 10^−4^) plants (Fig. 9A). There was no significant difference between the various tissues of the control plants in terms of F3′H and FLS genes expression (Fig. 9B,C). Treatment of Caper plants with 150 μM MeJA increased the expression of F3′H and FLS genes in all tissues compared with their control, but caused the highest expression of these genes (6.08 × 10^−3^ and 5.58 × 10^−3^, respectively) in young leaves (Fig. 9B,C). Also, the F3′H and FLS genes expression significantly was increased in all tissues of plants treated with 100 mgL^−1^ SA. Among the tissues of plants treated with SA, the highest expression of the F3′H and FLS genes were observed in the open flower (4.97 × 10^−3^) (Fig. 9B) and fruit (1.22 × 10^−2^) (Fig. 9C), respectively. Among the four genes studied, the expression of RT gene in different tissues of the control plants was higher than other genes (Fig. 9D). This gene showed the highest expression in the fruit (5.06 × 10^−3^) and leaf (4.17 × 10^−3^) tissues of the control plants (Fig. 9D). The application of MeJA and SA treatments on Caper plants significantly increased the expression of this gene in all of the plant tissues (except for the open flowers) compared with the control (Fig. 9D). The highest increases in the expression of the RT gene were observed in the leaves of the treated plants with MeJA (3.07 × 10^−2^) (Fig. 9D).

## Discussion

In the present study, two partial cDNA of rutin biosynthetic pathway, specifically 4CL, and RT, were isolated from *C.spinosa* for the first time and also a short segment of each F3′H and FLS genes was found from PRJNA311285 *C. spinosa* transcriptome sequencing project. As shown in Table 1, almost all of the four genes, identified in this study, showed the highest similarity to the same genes in the plants of the brassicaceae family or brassicales order. Also, phylogenetic analysis for RT amino acid sequence from *C. spinosa*, showed the highest similarity between this protein with other UDP-glycosyltransferase proteins from Brassicaceae family. Due to the fact that Caper is also a plant of brassicales order, this result can confirm the identification of these genes in this plant. Similar to these results were obtained from the comparison of amino acid sequences of enzymes associated with rutin biosynthesis in different plants with *A. thaliana*^23^, *F. esculentum*^2^, and *F. tartaricum* Gaertn^24^, Also, by comparing the sequence obtained in this study with two genes of RT and UFGT from different plant species such as *G.hirsutum*, *G.max* and *A.thaliana*, we proved that the identified sequence is a RT gene, a final glycosylation step in rutin biosynthetic pathway that rhamnosylate the glucose moiety of isoquercitrin^10^. Also some members of rhamnosyltransferases, which catalyze rhamnosylation of the sugar moiety of flavonoid glycosides, have been characterized in other plant species such as *Citrus sinensis* and *Citrus maxima*^25,26^, *G. max*^27^, *Lobelia erinus*^28^, and *F. esculentum*^10^.

Although the effects of SA and MeJA have been investigated as two stimuli for increasing flavonoids in many plant species, but there are no reports regarding the effects of these two chemical compounds on rutin accumulation as well as the expression of rutin biosynthetic pathway genes in Caper plants. The results showed that rutin content in Caper leaves increased significantly, 24 h after treatment with 100 mgL^−1^ SA, and this increase in rutin level was maintained when Caper plants treated with 150 mgL^−1^ SA. SA treatments of 50 mgL^−1^ had no significant effect on rutin content of Caper leaves. These results indicate that high levels of SA can stimulate the rutin biosynthetic pathway enzymes in the Caper and lead to increased rutin accumulation in the leaves of this plant. In accordance with the results of this study, other researchers^24^ showed that the application of 100 mgL^−1^ salicylic acid similarly increased the amount of rutin in *F. tataricum* leaves. Also, other researchers^29^ showed that treatment of buckwheat sprouts with 100 µM SA for 12–48 h significantly increased the amount of rutin in *F. esculentum* leaves. In the present study, all of the four genes related to rutin biosynthesis were up-regulated 24 h after treatment with different concentrations of SA (Fig. 8), indicating that SA can activate the expression of these genes, and thereby promote rutin biosynthesis. However, the highest expression levels for all genes were obtained when 100 mgL^−1^ SA was used as treatment. Also, the gene expression level of RT gene under 100 mgL^−1^ SA-treated conditions was the highest of any of the genes studied. This result indicates that RT, as the last step pathway gene of rutin biosynthesis, is more affected by SA treatment than the earlier steps in rutin biosynthesis. Our results indicate that the regulation by SA of the expression of rutin biosynthesis genes in *C.spinosa* plants occurs at the transcriptional level, and this is consistent with results in *F. esculentum*^29^ and *F. tataricum*^24^.

In the present study, the rutin concentration of MeJA-treated leaves increased as compared with the control, and peaked at 150 μM MeJA and again significantly decreased in 200 μM MeJA treatment. This result is in agreement with other studies which previously showed that MeJA is a good stimulant for increasing rutin biosynthesis^29,30^. Previous researchers^31^ reported the enhanced production of phytochemical in buckwheat sprouts treated with MeJA, that lead to an increase in the phenolic compound content and antioxidant activity of these sprouts. MeJA is a signaling molecule involved in plant growth, development and defense^32^ that can induce flavonoid biosynthetic gene expression and has also been found to enhance flavonoid accumulation in other plant species such as *Coleus forskohlii*^33^, *Salvia miltiorrhiza*^34^ and Satureja khuzistanica Jamzad^35^,

Our results indicated that MeJA influence the expression of four rutin biosynthesis genes (4CL, F3′H, FLS and RT) in Caper treated leaves than control plants. All studied genes were up-regulated by MeJA treatments which might be connected to rutin accumulation as well. The expression level of 4CL gene was significantly increased in Caper leaves following all MeJA treatments. The largest increase was observed following 100 and 150 μM MeJA treatments, where 4CL expression levels were 2.56 and 3.70 times that of control, respectively. Consistent with this result, the expression levels of 4CL were 2.43 times that of the control, in *F. esculentum* leaves treated with 100 μM MeJA^29^. Among the studied genes, the highest levels of expression was related to F3′H gene in leaves treated with 150 μM of MeJA, which were 36.73 times higher than expression of this in control plants, respectively. Also, the application of 150 and 100 μM of MeJA increased the expression of the rutin biosynthetic pathway genes of FLS (19.19 and 18.2 fold, respectively), and RT (3.8 and 5.3 times, respectively), as compared to the expression of these genes in the control plants. Results showed that the expression pattern of 4CL, F3′H, FLS and RT genes coincided well with the increase of rutin in 100 and 150 μM MeJA-treated plants (Fig. 4B) and the expression of all four genes were closely related to rutin biosynthesis. In agreement with these results, MeJA has been identified as a predominant inducer of global genes involved in secondary metabolite pathways in various plants, such as *Ocimum basilicum*^36^, *Tanacetum parthenium*^18^ and *S. khuzistanica* Jamzad^35^. All the four genes related to rutin biosynthesis investigated in this study, were upregulated by MeJA and SA treatments. These results suggest that MeJA and SA may play a role as a signaling factor in the rutin biosynthetic pathway.

Rutin content analysis showed significant variation among various tissues of *C.spinosa*. In the control plants, the highest amount of rutin was observed in young leaves and then significantly decreased in flower buds and increased again in open flowers. The lowest amount of rutin was observed in the fruits. On the other hand the biosynthesis and accumulation of rutin showed a zig-zag pattern during the developmental stages of *C. spinosa*. In accordance with these results, other researchers also showed that the highest amount of rutin is accumulated in Caper leaves, and the least amount is present in fruits^9,37^. Also, other studies reports^3,38^ a difference in the rutin levels at various developmental stages of buckwheat (from seed germination to mature seed formation). Variation in the amount of rutin (0.67% in leaves, 0.55% in flowers, and 0.018% in green stems) was also reported in American elderberry plant^7^.

Compared to control, the amount of rutin was increased up to 13.13 folds in the young leaves of Caper plants treated with 150 μM MeJA. The increase in rutin levels was also significant in open flowers of the treated plants with MeJA, while this treatment caused a slight increase in the rutin of buds and fruits. In contrast, SA treatment (100 mgL^−1^) only increased the rutin content in young leaves, and did not result in a significant increase in rutin of other tissues compared to their controls. This indicates that probably MeJA has stronger effect on rutin biosynthesis compared with SA. It has been also reported that MeJA was a better elicitor than SA for the production of rosmarinic acid in *C. forskohlii* hairy root cultures^13^, and also parthenoide production in *T. parthenium* leaves^18^. As described in a previous report^39^. the efficacy of MeJA and SA compounds in increasing plant secondary metabolites depends on many factors such as the concentration and mode of application of these compounds, the endogenous level of these compounds in plant, the type of tissue to be treated, as well as the developmental stage of the plant^40^. Taken together, the results of these studies confirm that biosynthesis and accumulation of secondary metabolites are usually tissue and developmental stage-specific in response to various environmental stimuli, biotic and abiotic such as MeJA and SA^39^.

In this study, gene expression was also compared in different tissues of control Caper plants and plants treated with 150 μM MeJA and 100 mgL^−1^ SA. The gene transcripts for all of the enzymes of the rutin biosynthetic pathway were expressed in all tissue of Caper plant. Of the four genes studied, three genes of 4CL, F3′H, and FLS were expressed at low levels in all tissues of the control Caper plants with no significant difference between their expression levels among various tissues. This was found consistent with previous report where F3H, F3′H, FLS1 and FLS2 genes were expressed at low levels in the various tissues of the buckwheat^41^. The RT gene showed the highest expression in fruits and leaves of control Caper plants, while the lowest expression of this gene was observed in open flowers of the control plants. In contrast, the concentration of rutin, was higher in the leaves and open flowers than in the buds and fruits. In other words, there was an inverse relationship between RT gene expression in buds, open flowers and fruits, and rutin accumulation in these tissues. This inverse relationship between the expression of flavonoid biosynthesis genes and the accumulation of their products has also been observed in Common Buckwheat (*F. esculentum*)^2^ and *A. thaliana*^42,43^ that may be due to the transport of flavonoids between different tissue of the plant^2,42–45^. On the other hand, as reported by earlier researchers^46^, the amount of rutin in different tissues of the caper plant can change during the day and night depending on the temperature and light intensity.

As shown in Fig. 9, MeJA and SA treatments increased the expression of all studied genes in all tissues of the Caper plant compared to their control. While the increased expression of these genes under the MeJA treatment resulted in a significant increase in rutin accumulation in the leaves and open flowers but resulted to a slight increase in the amount of rutin in the buds and fruits. Also, although SA increased the expression of all genes in all tissues (except the 4CL gene in leaf tissue), only the rutin content was significantly increased in leaves compared with control, while rutin contents in the open flowers and fruits were slightly affected by this treatment. It indicates that different flavonoid biosynthetic gene members of a family may respond differentially to external stimuli and play distinct roles in different tissues. In concordance with these results, previous reports^34^ also showed that the flavonoids biosynthesis related-genes of *S. miltiorrhiza* were expressed in a tissue-specific manner upon treatment with MeJA.

In the present study, the rutin content significantly declined in fruits of control and treated-Caper plants despite the increased expression of the related genes in this tissue. This suggests that probably a regulatory mechanism declines rutin content through conversion to other compounds. In previous studies, the reduction of rutin content in fruits has also been reported^36,47,48^. The higher rutin contents in the leaves, buds and open flowers compared to the fruits, may be due to the active phenolic synthesis in these tissues, which act as defense and protective agents. Previous studies^48^ reported that as the plant grows to maturity stages, some chemical or enzymatic degradation of the glycosides may occur, and the final products may be reused in another metabolic pathway. It is also possible that, with the development of flower buds to mature fruits, phenolic synthesis is directed towards the formation of other flavonoids, such as anthocyanins. Also, other researchers^49^ by studying the pattern of flavonoid accumulation in the *Berberis Buxifolia* suggested that reduction in flavonoid contents during ripening can be attributed to the degradation of flavonoids and their utilization in the biosynthesis of other compounds and/or association with other cellular compounds by stable covalent links.

In conclusion, we identified and isolated the partial cDNAs of four genes (4CL, F3′H, FLS and RT), encoding rutin biosynthesis enzymes in *C. spinosa*, for the first time. Our results showed that treatment with different concentrations of abiotic elicitors such as MeJA and SA lead to differential up–regulation of four genes involved in rutin biosynthesis in Caper leaves. The accumulation patterns of rutin coincided with the expression pattern of these genes indicating that these genes play important roles in rutin biosynthesis in Caper plants. In addition, the differential expression levels of these genes and the amounts of rutin were examined in different tissues of Caper plants treated with 150 µM MeJA and 100 mgL^−1^ SA. The results demonstrate that exogenously applied MeJA or SA differentially influenced the rutin accumulation and also expression level of related genes in different tissues of Caper. Among the various tissues, the highest contents of rutin were observed in leaves of Caper plant treated with 150 µM MeJA, is most likely further positively related to RT gene expression as the end committed step of rutin biosynthesis. This study will be useful in expanding our understanding of the molecular basis of the flavonoid accumulation in *C.spinosa*, as an important source of rutin and developing strategies to increase the yield of medicinal compounds in this valuable plant species.

## Materials and Methods

### Plant material and growth conditions

This experiment was conducted at the Faculty of Agriculture, Bu-Ali Sina University, Hamedan, Iran, from June 2017 to October 2018. The plant material used in this experiment was harvested from wild caper plants grown in Isfahan province at vegetative growth (June 2018), when the plants had only root, stem and leave parts and fresh fruiting stage (August 2018), when the plants had root, stem, leave, bud, flower and fresh fruits. Sampling was done once a day (10 AM) for each development stage. The plants were grown under natural conditions such as light (~ 450–600 µmol m^−2^ s^−1^) and natural temperatures conditions (ranged from 29 to 32 °C during the day and from 18 to 22 °C at night). The soil of research area was a mixture of clay with very thin veins of sand and also scattered lime in the soil profile.

### Experiment I: treatment of Caper plants with different concentrations of SA and MeJA

Both SA (MERCK, Germany) and MeJA (SIGMA-ALDRICH) solutions were filter sterilized with a filter membrane (0.22 μm, MILLIPORE). Caper plants at vegetative stage (June 2018) were treated with SA at final concentrations of 50, 100 and 150 mgL^−1^ and MeJA at final concentrations of 10, 100, 150 and 200 μM). For each treatment, three uniform plants of Caper were sprayed with 1000 ml of SA or MeJA solutions until runoff. Control plants were sprayed with 1000 ml distilled water. After 24 h, the leaves of treated and control plants were harvested and then immediately frozen in liquid nitrogen and stored at −80 °C until further use.

### Experiment II: treatment of Caper plants with 100 mgL^−1^ SA and 150** μM** MeJA

In August 2018, when the Caper plants were at fresh fruiting growth stage, three plants that were used for 100 mg L^−1^ SA and 150 μM MeJA treatments in the first experiment were sprayed again with 1000 ml of SA (100 mg L^−1^) and MeJA solutions (150 μM) until runoff. The plants that were used in the first experiment as control were sprayed again with 1000 ml distilled water (pH 7) until runoff. Twenty four hours after initial treatments, various tissues of Caper plant including young leaves (terminal ones), buds (10–15 mm in length), open flowers (40–45 mm in length) and fruits (25–30 mm in length) were harvested from control and treated plants, treated with liquid nitrogen and kept at −80 °C until further use..

### Total RNA extraction, cDNA synthesis and gene identification

Total RNA was isolated from the frozen Caper plant samples by using the RNX-Plus Kit (CINNAGEN, Tehran, Iran) according to the manufacturer’s instructions. The harvested samples were ground using a sterilized mortar and pestle with liquid nitrogen and the quality of the extracted RNA was assessed by the A260/A280 absorbance ratio and 1% (w/v) agarose gel electrophoresis. For identification of the 4Cl and RT partial cDNAs, 3 μg of total RNA was reverse transcribed using the Viva 2-steps RT-PCR Kit (VIVANTIS, Malaysia). Gene-specific primer sets for 4CL and RT genes (Table 3) were designed by using the conserved sequences of known orthologous sequences in brassicales order. Also, the partial cDNA segments of F3′H and FLS genes were found in PRJNA311285 *Capparis spinosa* transcriptome sequencing project (Table 4). The partial sequences of 4CL and RT genes were sequenced by Bioneer Company (BIONEER Company, Korea). Sequence similarities of the PCR products were calculated with the Basic Local Alignment Search Tool for Nucleotides (BLASTN) (http://www.ncbi.nlm.nih.gov/BLAST). Finally, the amplicons sequences were submitted in NCBI database with GenBank accession numbers 4CL (MK301444) and RT (MK301445).

**Table 3:** Primers used to identify the 4CL and RT genes.

Primers	Sequences (5ʹ to 3ʹ)	Tm °C	Amplicon Size (bp) (partial)
4CL *F*	GCCAAACACATCGCTTATAACT	58	195
4CL *R*	TAGCCGTTGATCTCGCAGC		
RT *F*	TCGCCAACGAATTAGCCCAG	57	800
RT *R*	TCACTCGTTCTTCGAACCCC

**Table 4:** The partial cDNA segments of F3′H and FLS genes, were found from PRJNA311285 *Capparis spinosa* transcriptome sequencing project.

Genes	Partial sequences (5ʹ to 3ʹ)	Size (bp)
F3′H	CTTGGTAGTTATAAGCGATGTGTTTGGCTCCGGAGTTTGGGGGTCGGCTCGAGAAGTTGGCGTCATGGACCTTCAGGAACTGCTCCGCCACGGCCTTCGAC	101
FLS	CCTCATTCACCTCCCTGTAAGAGGGGGGGTTTTTGGGCCAGAATTCGTAGTTAACGCGAGAAGGCGGCCATATTCTATGGAACAGATGATCAACCCACGCC	101

### Phylogenetic analysis

A phylogenetic tree was created using the UPGMA method with 1000 bootstrap replicates. The tree was constructed with MEGA7 software using related UDP-glycosyltransferase amino acids sequences aligned with the RT amino acid sequence from *C.spinosa* L.

### Expression analysis of flavonoid (rutin) biosynthesis genes by qRT-PCR

Real-time PCR reactions were performed on a light cycler real time 480 system (ROCHE, Switzerland) using the SYBR premix Ex Taq II (TAKARA, Japan). The real time primers (Table 5) were designed using AlleleID software. Gene expression was normalized to that of the Ubiquitin gene as a housekeeping gene. The qRT-PCR reaction solution was composed of 10 μl of SYBR premix Ex Taq II, 0.3 μl of each primer (10 μM), 1 μl of cDNA, and 8.4 μl of RNase-free water in a total volume of 20 μl. The qRT-PCR protocol was as follows: initial denaturation for 30 s at 94 °C, followed by 40 cycles of denaturation for 5 s at 94 °C, annealing for 30 s at 52–56 °C, and elongation for 20 s at 72 °C. The annealing temperatures were 54 °C for Ubiquitin and FLS primers, 58 °C for 4CL, 52 °C for F3′H and 57 °C for RT primers. All qRT-PCR reactions were normalized using the Ct value corresponding to the Ubiquitin gene. Three biological replicates were used and two measurements were performed on each replicate. Normalized raw Ct values were used to compare results of control and treated samples using the 2^−∆∆Ct^ (Fold change) method^50^ in first experiment, while the 2^−∆Ct^ method^51^ was used for relative gene expression (RGE) measurements in second experiment.

**Table 5:** Primers used to qRT-PCR analysis.

Real-Time Primers	Sequences (5ʹ to 3ʹ)	Tm °C	Amplicon Size (bp)
4CL *F* 4CL *R*	TTCCATATTCACACAGAGAC CTTGACTGAAAGCACTGG	58	94
F3′H *F* F3′H *R*	GGTAGTTATAAGCGATGTGTTTG CGGAGCAGTTCCTGAAGG	52	84
FLS *F* FLS *R*	GCTGTAATCTTTGAAGGTGAAGG TCTTGTCGTCGGACCACTC	54	82
RT *F* RT *R*	TCCTCAACCAGACATCACCAAAC GCCAAACGCACAGAACACACC	57	94
UQ *F* UQ *R*	AAGACCTACACCAAGCCCAA AAGTGAGCCCACACTTACCA	54	196

### Extraction and HPLC analysis

Second set of control and treated plant samples were harvested at the same time and dried in dark at room temperature with a continuous air flow. Each sample was ground (5 gram) and suspended in methanol/water (80/20 v/v) solution in a 250 Erlenmeyer flask in orbital incubator shaker at 120 rpm for 3 days and subsequently incubated in an ultrasonic bath for 10 min. Following it, the samples were filtered with Whatman filter papers and the solvent was evaporated at 40 °C using rotary evaporator (HEIDOLPH, Germany). The residues were dried and the hydroalcoholic extract was scraped keep in a dark place. Twenty mg of each extract was dissolved in 2 mL methanol and was filtered through a 0.45 µm PTFE filters. Finally, 20 µL of the filtered extracts was injected into the HPLC column {Spherisorb ODS-2 (5 mgL^−1^) reversed phase 4.6 mm × 250 mm, SHIMADZU, Japan} for rutin measurement. Calibration curve was obtained by running multiple injections of different concentrations of standard rutin (SIGMA).

Two mobile phases, A and B were used: mobile phase A was 1% (v/v) acetic acid in water and mobile phase B was methanol. Solvent composition of gradient was as follows: 90% A and 10% B for the first 10 min, followed by 50% A and 50% B for the next 20 min, and finally 100% B for an additional 35 min. The flow rate was maintained at 1 mL min^−1^, and the detection wavelength (λ_max_) was 360 nm. The chromatographic peak of rutin was confirmed by comparing the retention time of reference standard, spectra of the samples and spike of standard. The quantitative analysis was performed with external standardization by measurement of the peak areas using the Agilent ChemStation (Figs. 2 and 3A–C). All samples were analyzed in triplicate.

### Data analysis

Sequence comparison was performed by BLAST Search in GenBank (http://www.ncbi.nih.gov). A completely randomized design (CRD) with three replications (three uniform plants) was used for the comparison of the rutin concentration and expression of genes (Fold change) in the first experiment (MeJA and SA treatments). A factorial experiment based on CRD design with three replications (three uniform plants) was used to compare rutin concentration and relative gene expression (RGE) in different tissues of Caper (Second experiment). In both experiments, three biological replicates and two technical replicates were used for qRT-PCR. All the data were statistically analyzed using SPSS statistical software (version 16). Multiple comparisons of means were carried out using Duncan’s multiple range tests (DMRT).

## Supplementary information

Supplementary information.
