# Supplementary material for: Identification and tissue-specific expression of rutin biosynthetic pathway genes in Capparis spinosa elicited with salicylic acid and methyl jasmonate
Source: Sci Rep. 2020 Jun 1;10:8884. doi: 10.1038/s41598-020-65815-2 (PMC7264309; doi:10.1038/s41598-020-65815-2)

Identification and tissue-specific expression of rutin biosynthetic pathway genes in *Capparis spinosa* elicited with salicylic acid and methyl jasmonate

Farzad Kianersi, Mohammad Reza Abdollahi, Asghar Mirzaie-asl, Dara Dastan, Faiza Rasheed

**Figure 6 A**


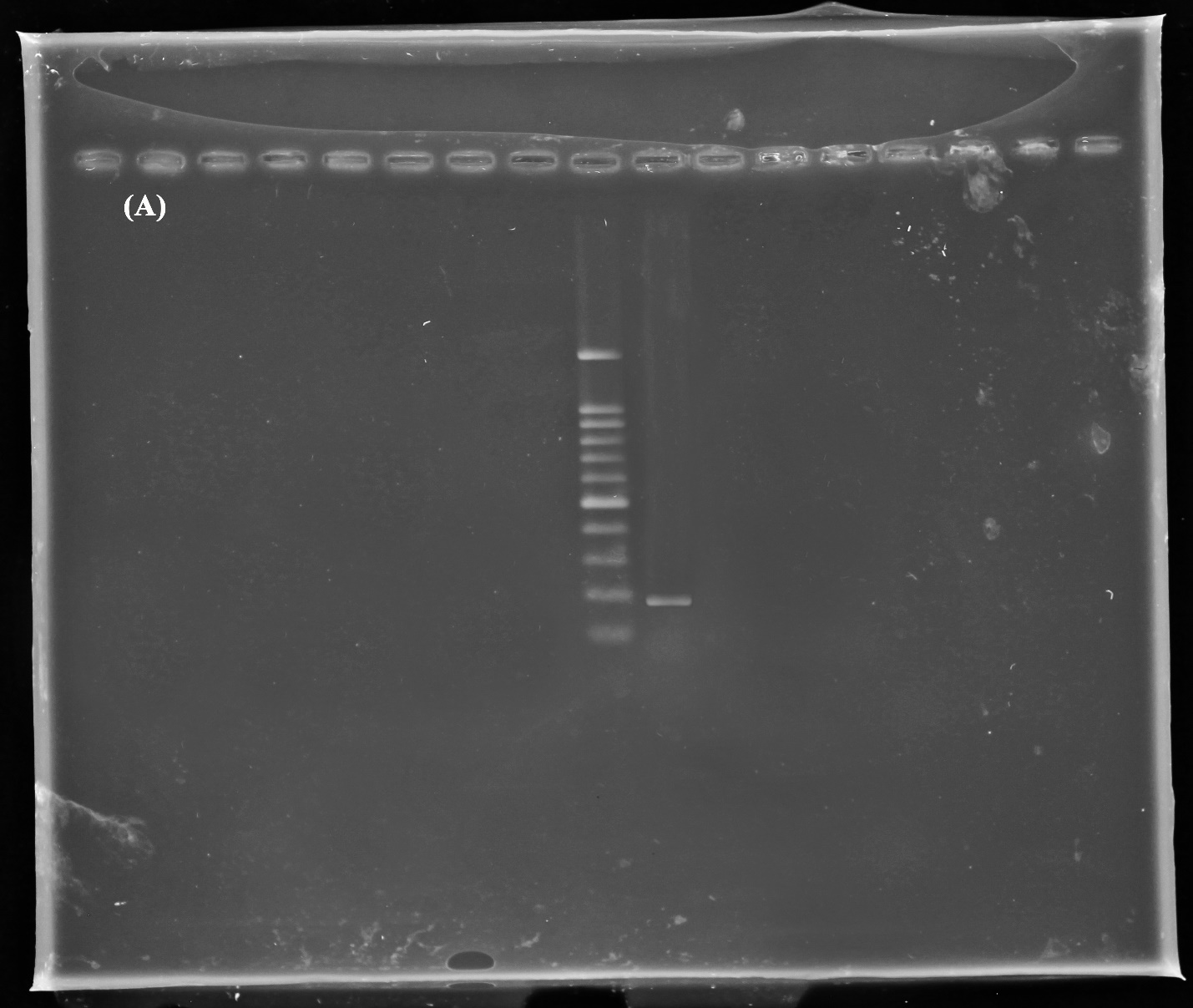


**Figure 6B**


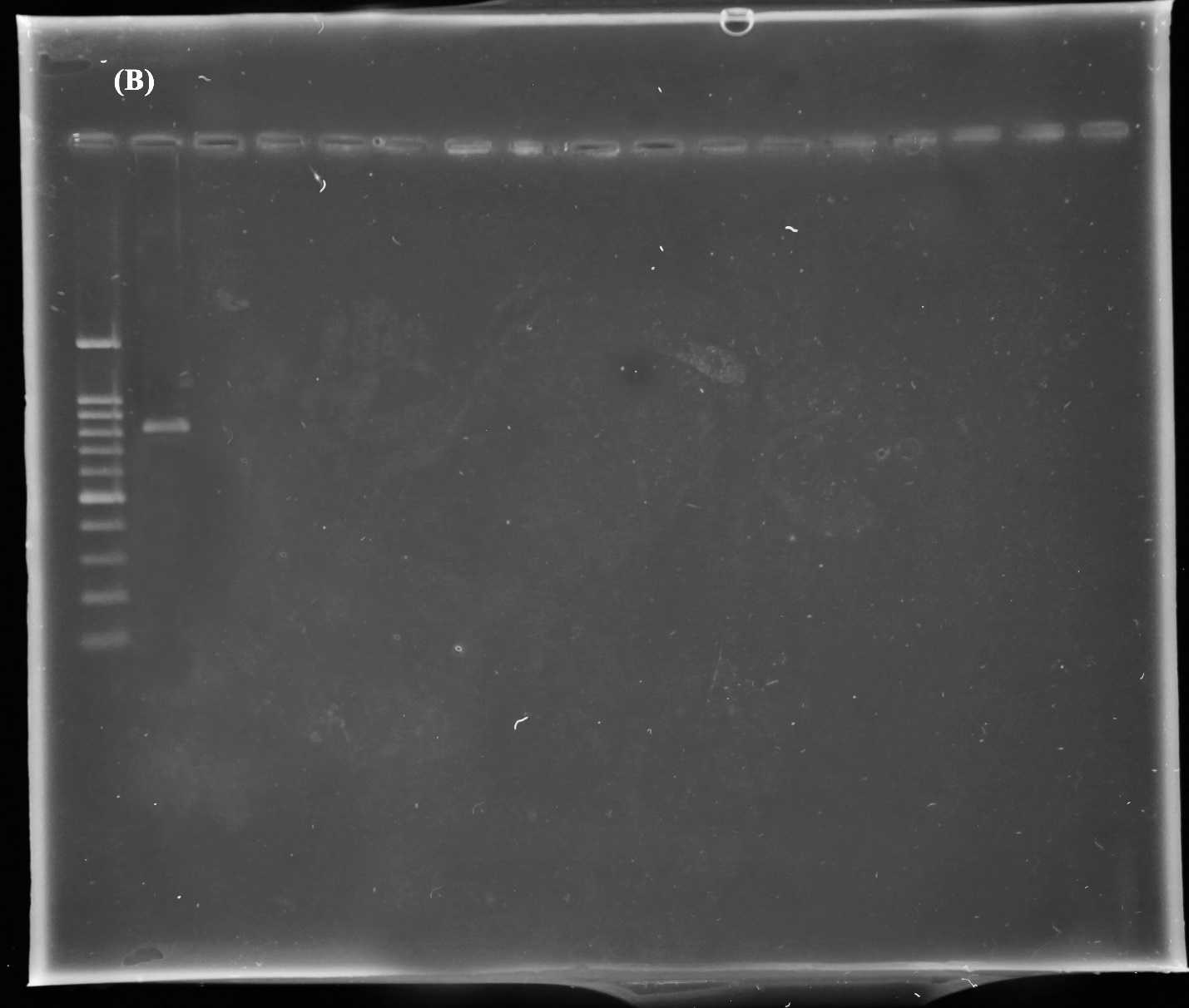

Supplement: Supplementary file 1 — Supplementary information. [file 41598_2020_65815_MOESM1_ESM.docx]
